# Colonic Necrosis in a 4-Year-Old with Hyperlipidemic Acute Pancreatitis

**DOI:** 10.1155/2016/9123163

**Published:** 2016-01-27

**Authors:** Tiffany J. Patton, Timothy A. Sentongo, Grace Z. Mak, Stacy A. Kahn

**Affiliations:** ^1^Department of Pediatrics, Section of Pediatric Gastroenterology, University of Chicago, Wyler Pavilion C-474, 5839 S. Maryland Avenue, MC 4065, Chicago, IL 60637, USA; ^2^Department of Pediatrics, Section of Pediatric Surgery, The University of Chicago Hospitals, 5841 S. Maryland Avenue, MC 4062, Chicago, IL 60637, USA

## Abstract

Here we report the case of a 4-year-old male with severe acute pancreatitis due to hyperlipidemia, who presented with abdominal pain, metabolic abnormalities, and colonic necrosis. This colonic complication was secondary to the extension of a large peripancreatic fluid collection causing direct serosal autodigestion by pancreatic enzymes. Two weeks following the initial presentation, the peripancreatic fluid collection developed into a mature pancreatic pseudocyst, which was percutaneously drained. To our knowledge, this is the youngest documented pediatric case of colonic necrosis due to severe pancreatitis and the first descriptive pediatric case of a colonic complication due to hyperlipidemia-induced acute pancreatitis.

## 1. Introduction

Colonic complications of pancreatitis, that is, necrosis, fistulae, and strictures, are uncommon in adults but are rarely reported in children [1]. Mechanistic theories of colonic involvement in acute pancreatitis include mesenteric ischemia, thrombosis/compression of mesenteric arteries, local enzymatic digestion, and disseminated intravascular coagulation [2]. An extensive PubMed search identified two reports of colonic necrosis associated with acute pancreatitis in children, one ascribed to hypotension and the other to direct extension of necrotizing pancreatitis in an immunocompromised patient [3, 4]. Here we present a child with previously undiagnosed hyperlipidemia who presented with colonic necrosis from an adjacent peripancreatic fluid collection associated with severe acute pancreatitis.

## 2. Case Presentation

A 4-year-old male with no known medical problems presented to a local emergency room with a 3-day history of decreased oral intake, nausea, nonbloody, nonbilious emesis, and abdominal pain. Laboratory assessment revealed lipemic serum with triglycerides of 7449 mg/dL (normal 30–149 mg/dL), anemia (hematocrit 19%), hyponatremia (sodium 129 mEq/L), metabolic acidosis (carbon dioxide 12 mEq/L), azotemia (blood urea nitrogen 73 mg/dL, creatinine 1.8 mg/dL), amylase 248 IU/L (normal 28–100 IU/L), and lipase 360 U/L (normal 13–60 U/L). A contrast abdominal CT scan demonstrated a large retroperitoneal mass appearing to arise from the left adrenal gland with normal pancreatic appearance. The patient was transferred to the University of Chicago Pediatric Intensive Care Unit for further management and diagnostic workup.

On admission the patient was febrile (39.4°C) and tachycardic (132 beats/min) with weight, height, and BMI at the 83rd, 33rd, and 97th percentiles, respectively. On physical exam his abdomen was distended with hypoactive bowel sounds and a palpable mass in the left upper quadrant; no xanthomata or lipemia retinalis was noted. The laboratory findings were comparable to the previous with the addition of amylase 79 U/L (normal 28–100 U/L), lipase 99 U/L (normal 11–65 U/L), and fasting triglycerides 6060 mg/dL.

**Figure 1:**
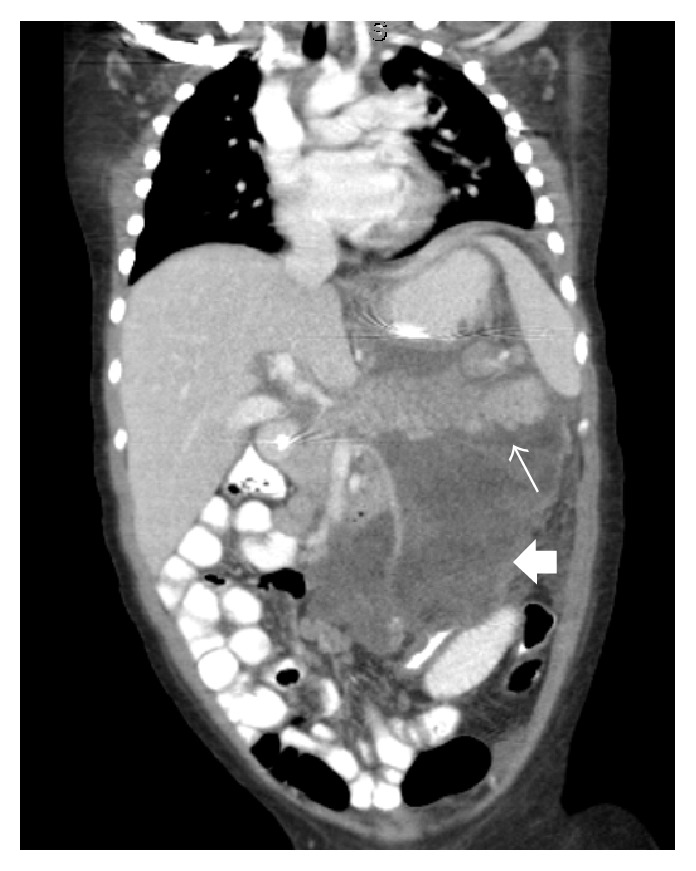
Abdominal CT (coronal view): 10 cm × 8.4 cm left-sided peritoneal mass (broad arrow) with significant inflammation of the pancreas (thin arrow).

Due to concern for malignancy, the patient underwent an extensive workup to rule out solid tumors. On repeat chest/abdominal CT scan, a diffusely enlarged pancreas without areas of necrosis or calcifications was noted. Additionally, the mass was described as a large infiltrative low density lesion extending from the left retroperitoneum around the pancreas, bilateral renal spaces, down to the perirectal area without obvious metastatic lesions within the lungs or abdomen (Figure 1). The patient underwent an exploratory laparotomy with tissue histology to better characterize the origin of the mass. Intraoperatively, a significant amount of ascites and the retroperitoneal mass were identified in the left paracolic gutter with necrotic extension to the omentum and onto the left colon at the splenic flexure. The mass was also noted within the lesser sac, anterior to the pancreas, and extending into the pelvis. A large portion of the retroperitoneal mass and adherent omentum was resected, in addition to the necrotic portion of the splenic flexure, and a diverting transverse colostomy was performed. The mass pathology revealed extensive fat necrosis and reactive inflammation of the omentum without any identifiable tumor cells. The resected bowel segment had extensive serosal fat necrosis and reactive inflammatory changes, but viable mucosa and muscular wall with no perforation on pathologic examination. Additionally, bone marrow biopsies and infectious studies were negative. Two weeks later, a repeat abdominal CT scan revealed a large mature pseudocyst in the site of the former fluid collection, which was then percutaneously drained by interventional radiology (amylase level of 173 U/L).

We concluded that the patient had undiagnosed type I familial hypertriglyceridemia, presenting initially as chylomicronemia syndrome, resulting in acute pancreatitis with a large peripancreatic fluid accumulation causing colonic necrosis due to direct pancreatic enzyme serosal digestion. The patient was initially managed with total parenteral nutrition and kept NPO (nil per os) with subsequent advancement to a low-fat diet and colonic reanastomosis following a 3-month period. To date his hypertriglyceridemia has been controlled with a low-fat diet supplemented with omega-3 fatty acid ethyl esters.

## 3. Discussion

Colonic complications, such as necrosis, fistulae, and strictures, have been reported in 15% of adults with severe pancreatitis [1]. To date only two reports with differing etiologies have been published describing colonic necrosis associated with acute pancreatitis in children [3, 4]. Current literature describes three separate mechanisms for the etiology of pancreatitis associated colonic injury. First, the direct spread of pancreatic enzymes and peripancreatic inflammatory tissue can involve the colonic serosa, causing local inflammation and fat necrosis. Second, thrombosis/compression of the mesenteric and submucosal vessels can lead to infarction of the mucosa and deeper layers of the colon [4, 5]. Third, hypotensive episodes in severe acute pancreatitis can lead to a “low flow state” causing ischemia of the colon particularly at the splenic flexure at the junction of the middle and left colic arteries [2]. Given the related findings within the present case, we suspect that the direct extension of peripancreatic fluid and its pancreatic enzyme content were responsible for the adjacent colonic necrosis in our patient.

In adults, pancreatitis associated colonic necrosis may be diagnosed at a median of 25 (range 1–55) days following symptomatic onset and has a mortality rate of 54% [1]. While the pediatric mortality rate for pancreatitis associated colonic necrosis is unknown, it is generally accepted that the mortality rate in pediatric acute pancreatitis (~2–11.1%) is often due to concurrent systemic illness [6]. In the present case, colonic necrosis was discovered intraoperatively 8 days following initial symptomatic onset. In all cases, colonic resection with a diverting stoma is the standard therapeutic management of colonic necrosis, as was described in our case [1].

The association of hyperlipidemia and acute pancreatitis has been well described and accounts for ~6% of acute pancreatitis episodes [5, 7]. Acute pancreatitis is associated with increased serum lipids in 50% of patients, while a triglyceride level > 1,000 mg/dL can precipitate an episode of acute pancreatitis [8]. The pathophysiology of hypertriglyceridemia-induced pancreatitis is not well established. However, it is thought to occur when pancreatic lipase digests triglycerides within pancreatic capillaries causing chylomicrons to occlude these vessels leading to pancreatic ischemia and activation of trypsinogen by free fatty acids [8]. Theoretically, the resultant hyperviscosity from chylomicrons may impair circulatory flow within smaller splanchnic vessels, predisposing to colonic complications (i.e., colonic necrosis, ischemia, and perforation). However, we do not believe that this was the etiology in our patient due to a lack of necrosis within the mucosal and intramural layers of the colonic wall.

Chylomicronemia syndrome, as identified in our patient, is defined as a plasma triglyceride level > 1000 mg/dL accompanied by one or more of the following: eruptive xanthoma, lipemia retinalis, abdominal pain, acute pancreatitis, and/or hepatosplenomegaly [9]. Familial hypertriglyceridemia is the most common primary cause of chylomicronemia in adults. Treatment of chylomicronemia involves lifestyle modification with increased physical activity, weight loss, and a low-fat diet (i.e., reduction of saturated fat to <7% of total calories). Medical therapy includes fibrates and/or n-3 polyunsaturated fatty acids, both of which are capable of reducing triglycerides by 50% [9]. For severe cases of chylomicronemia (triglycerides > 500 mg/dL and abdominal pain or pancreatitis), inpatient therapy is preferred with NPO, intravenous hydration, low dose insulin and/or heparin infusions, and gradual advancement to a low-fat diet. Accordingly, our patient remained NPO for two weeks prior to advancing to a low-fat diet with a corresponding decrease in serum triglycerides. Insulin and heparin infusions were not instituted in our patient due to a relatively rapid reduction in triglyceride levels while being NPO.

Although colonic complications are far more prevalent in the adult population, pediatricians need to be aware that they can occur in children with pancreatitis. Additionally, since the association between hyperlipidemia and pancreatitis is well established, it is important for clinicians to consider hyperlipidemic pancreatitis and its complications when faced with lactescent serum samples (and/or fasting serum triglyceride levels > 1000–2000 mg/dL) and abdominal pain even in the presence of normal or mildly elevated serum amylase and lipase. It is known that lipemia commonly interferes with the processing methods of various laboratory tests, potentially rendering test results inaccurate and difficult to interpret [10].

To our knowledge, this is the first and youngest pediatric report of colonic necrosis due to direct enzymatic contact with a peripancreatic fluid collection in acute pancreatitis secondary to hyperlipidemia. Several key features of this case should be noted. First, the patient presented with extremely high triglyceride levels although there was no significant history of hyperlipidemic symptoms. Second, absence of significantly elevated serum amylase and lipase in the setting of hyperlipemic serum may obscure the diagnosis of pancreatitis [11, 12]. Therefore in this setting, subtle but ongoing gastrointestinal symptoms including vomiting and abdominal pain warrant further evaluation including a workup for pancreatitis. Finally, although this presentation was initially concerning for malignancy (i.e., neuroblastoma, Wilms' tumor, hepatoblastoma, and lymphoma), in addition to an intraperitoneal abscess, the differential diagnosis of a retroperitoneal mass should always include complications of pancreatitis and pancreatic pseudocyst.
